# Five-year patient-reported outcomes after fixed-bearing medial UKA with broad patient selection

**DOI:** 10.1186/s42836-026-00380-z

**Published:** 2026-04-10

**Authors:** Emily M. London, Damian A. Bull, Katy L. Mason, Tahir Idrees, Jacobus H. Müller, Harrogate Knee Study Group, Caroline Bennett, David J. Duffy, Ashim Mannan, Richard W. D. Pilling, Mo Saffarini, Caroline Sköld, Nick J. London

**Affiliations:** 1 Mid Yorkshire Teaching NHS Trust, Wakefield, WF1 4DG United Kingdom; 2 Harrogate District Hospital, Harrogate, HG2 7SX United Kingdom; 3 The Duchy Hospital, Circle Health Group, Harrogate, HG2 0HF United Kingdom; 4 Leeds Teaching Hospitals, Leeds, LS9 7TF United Kingdom; 5 ReSurg SA, 1260 Nyon, Switzerland; 6 Carnegie School of Sport, Leeds Beckett University, Leeds, LS6 3QS United Kingdom

**Keywords:** Unicompartmental knee arthroplasty, UKA, Fixed-bearing, PROMs

## Abstract

**Purpose:**

Evaluate the impact of patient age, body mass index (BMI), medial/central patellofemoral arthritis, and anterior cruciate ligament (ACL) deficiency on five-year patient-reported outcome measures (PROMs) of fixed-bearing medial unicompartmental knee arthroplasty (UKA).

**Methods:**

A consecutive group of 229 patients (240 knees) received fixed-bearing medial UKA. At minimum two (*n* = 231 knees) and five years (*n* = 221 knees), patients completed the Oxford Knee Score (OKS), the EuoQol-5D (EQ-5D), Knee injury and Osteoarthritis Outcome Score Physical Function Short Form (KOOS-PS), University of California, Los Angeles (UCLA) activity score, Forgotten Joint Score (FJS), and their level of satisfaction. Sub-group analyses compared PROMs in patients based on: (i) Grade III/IV vs. Grade 0–III medial/central patellofemoral arthritis, (ii) ACL deficiency vs. intact ACL, (iii) age groups (< 50, 50–59, 60–69, 70–79, > 80), and (iv) BMI categories (< 30, 30–35, 35–40, ≥ 40).

**Results:**

Satisfaction rates remained consistent at the 2- and 5-year follow-up points, with 96% being satisfied or very satisfied. The OKS, EQ-5D, KOOS PS, or FJS-12 were not statistically significantly different between 2 and 5 years. Five-year UCLA activity scores differed significantly across age groups (50–59 vs 80 + (MD = 1.5; *p* = 0.027), 60–69 vs 70–79 (MD = 0.9; *p* = 0.014), and 60–69 vs 80 + (MD = 1.7; *p* = 0.004)), and between patients with a BMI < 30 vs ≥ 40 (MD = 2.3; *p* = 0.045). These findings were supported by multivariable regression, which showed that increasing age and higher BMI were independently associated with worse UCLA activity scores. Grade III/IV patellofemoral arthritis was associated with worse EQ-5D scores but was not associated with worse OKS, UCLA, KOOS-PS, or FJS-12. ACL deficiency was associated with higher KOOS-PS scores.

**Conclusion:**

Five-year outcomes following fixed-bearing medial UKA demonstrated high patient satisfaction, unchanged from two years. Although older age was associated with lower activity and higher BMI (> 40) with worse function, the effect sizes were small and not clinically meaningful. Patellofemoral arthritis and ACL deficiency had no negative functional impact. Therefore, age, BMI, patellofemoral arthritis, and ACL status should not be considered contraindications; instead, broad selection criteria for fixed-bearing UKA are supported.

## Introduction

Unicompartmental knee arthroplasty (UKA) is a less invasive alternative to total knee arthroplasty (TKA) and has gained popularity due to its better functional outcomes and bone conservation, as well as reduced surgical risk (e.g., infections, thromboembolic events) and more favourable options for revision [1–3]. The 2025 National Joint Registry reported that Kaplan Meier estimates for cumulative revision of cemented fixed-bearing UKA were 3.18% (95% confidence interval (CI), 3.03% to 3.35%) at 5 years and 6.21% (95% CI, 5.92 to 6.50%) at 10 years (www.njrcentre.org.uk). Additionally, among various designs and brands, the fixed-bearing UKA (with cemented metal-backed tibial component) demonstrates exceptional survivorship at and beyond product lifetime: 7.90% (15 years) and 4.92% (10 years) for the Physica ZUK, and 1.98% for the newer Persona Partial Knee at 5 years.

In 1989, Kozinn and Scott [4] proposed very narrow criteria for UKA, limiting its use to a select group of knee arthroplasty patients, a guideline many surgeons still follow 35 years later, often resulting in few or no UKA procedures. The hesitation of some surgeons to consider UKA as a treatment option arises from concerns about lower survivorship and patient-reported outcomes if an arthritic joint surface is left untreated [5]. Technological advancements, improved understanding of knee biomechanics, and refined surgical techniques have challenged the Kozinn and Scott criteria [6]. While age and activity levels are no longer widely considered contraindications [7], the influence of body mass index (BMI) [8–11], patellofemoral joint arthritis [11, 12], and anterior cruciate ligament status [13] on UKA outcomes remains controversial and inconsistent.

The authors previously reported outcomes of fixed-bearing medial UKA at a 2-year follow-up. They concluded that they are satisfactory in knees with substantial medial/central patellofemoral arthritis or functionally stable anterior cruciate ligament (ACL) deficiency [14]. The purpose of this study was to report the outcomes of the same cohort at a 5-year follow-up and to determine whether, in addition to medial/central patellofemoral arthritis or ACL deficiency, patient age and body mass index (BMI) affect the outcomes of fixed-bearing medial UKA. The hypothesis was that PROMs would be unaffected by substantial medial/central patellofemoral arthritis, functionally stable ACL deficiency, age, or BMI.

## Materials and methods

### Study design and setting

The authors retrospectively examined a consecutive group of 229 patients (240 knees) who received fixed-bearing medial UKA (Persona Partial Knee, Zimmer Biomet, Warsaw, USA) between February 2017 and February 2019 under the care of the senior surgeon (NJL), who has an annual caseload of approximately 180 UKAs representing more than 60% of his primary knee arthroplasty practice. The authors previously reported outcomes of the same cohort at 2-year follow-up [14]. All patients were referred and provided informed consent for collecting and using their data. This study was conducted in accordance with the principles of Good Clinical Practice and the Declaration of Helsinki. Approval was obtained from the institutional review board (Research Ethics Committee reference: 21/NW/0120), and all patients provided informed consent.

The inclusion criteria were tibiofemoral arthritis limited to the medial compartment, confirmed by clinical and radiographic examination. Patients presenting with anterior knee pain were assessed by history and physical examination (i.e., loaded deep flexion, stair climbing, and descending) to identify those with symptomatic patellofemoral arthritis. These patients underwent further radiographic assessment using skyline patellofemoral radiographs or magnetic resonance imaging (MRI) to include those with medial or central arthritis and exclude those with severe lateral arthritis. Patients with ACL deficiency (i.e., identified from their history or clinical examination) were questioned regarding symptoms of rotational instability (very rare in an arthritic population), and those with no complaints of rotational instability (functionally stable ACL deficiency [15]) were included in the study. No patients were excluded due to rotatory instability. All ACL-deficient knees included in the study were functionally stable, as confirmed by preoperative clinical assessment and intraoperative testing, which involved passing a hook around the native ACL and applying firm traction [15]. Exclusion criteria were based on the National Institute for Health and Care Excellence (NICE) guidelines: > 15° varus deformity, lateral tibiofemoral arthritis, or symptomatic lateral patellofemoral arthritis.

Of the initial cohort of 229 patients (240 knees), 12 patients (12 knees) died from causes unrelated to their UKA, two patients (2 knees) were lost to follow-up, and one patient (1 knee) refused further participation in the study. Four patients (4 knees) had a revision procedure:

- UKA was revised to TKA due to a periprosthetic fracture at 11 months.
- The tibial component was revised due to tibial loosening at 26 months.
- UKA was revised in another unit to TKA due to unexplained pain at 31 months.
- UKA was revised to TKA due to increasing lateral tibio-femoral arthritis at 35 months.

**Fig. 1:**
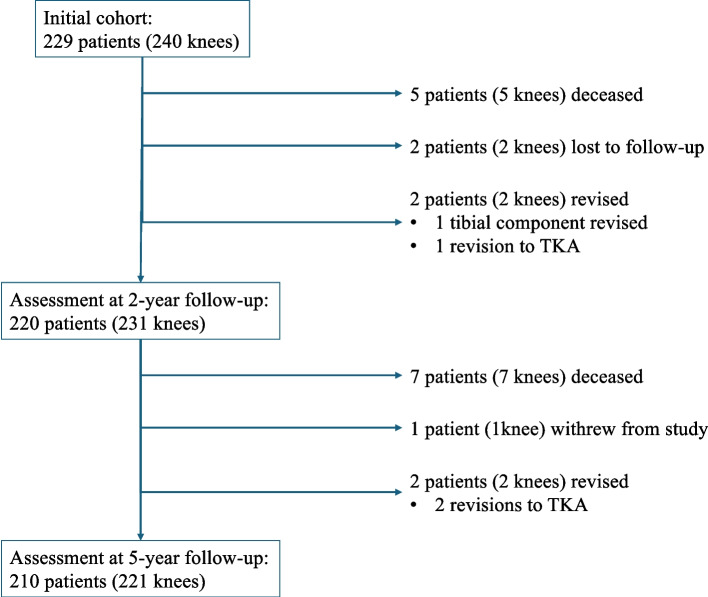
Flowchart of the study cohort

The remaining 210 patients (221 knees) were assessed at a minimum follow-up of 5 years (Fig. 1; Table 1).

**Table 1:** Patient preoperative demographic and clinical characteristics

Characteristic	*n* (%)	Mean ± SD	Range	Median (IQR)
**Age (years)**		65.6 ± 9.8	41–89	66 (58–72)
**BMI (kg/m**^**2**^**)**		29.2 ± 4.6	20–47	29 (26–32)
**Men**	128 (55%)			
**Right knees**	123 (56%)			
**Bilateral UKA (patients)**	11 (5%)			
**Varus deformity**				
Degree (°)		6.5 ± 2.0	2–12	7 (5–8)
Fully correctable	213 (96%)			
Partially correctable	8 (4%)			
**Knee range of motion (°)**		120.6 ± 9.6	85–140	120 (120–125)
**Pain location**				
Medial only	216 (98%)			
Medial + Anterior	4 (2%)			
Lateral + Anterior	1 (< 1%)			
**Outerbridge assessment**				
**Tibiofemoral medial compartment**				
None	—			
Grade I	—			
Grade II	1 (< 1%)			
Grade III	6 (3%)			
Grade IV	214 (87%)			
**Tibiofemoral lateral compartment**				
None	205 (93%)			
Grade I	4 (2%)			
Grade II	11 (5%)			
Grade III	—			
Grade IV	1 (< 1%)			
**Patellofemoral lateral facet**				
None	175 (79%)			
Grade I	7 (3%)			
Grade II	17 (8%)			
Grade III	10 (5%)			
Grade IV	12 (5%)			
**Patellofemoral central/medial facet**				
None	57 (26%)			
Grade I	5 (2%)			
Grade II	39 (18%)			
Grade III	36 (16%)			
Grade IV	84 (38%)			
**ACL status**				
Intact	201 (91%)			
Deficient	20 (9%)			

Empty cells (—) indicate data that were not applicable

*Abbreviations:* BMI, body mass index; UKA, unicompartmental knee arthroplasty; ACL, anterior cruciate ligament; SD, standard deviation; IQR, interquartile range

### Surgical technique

**Fig. 2:**
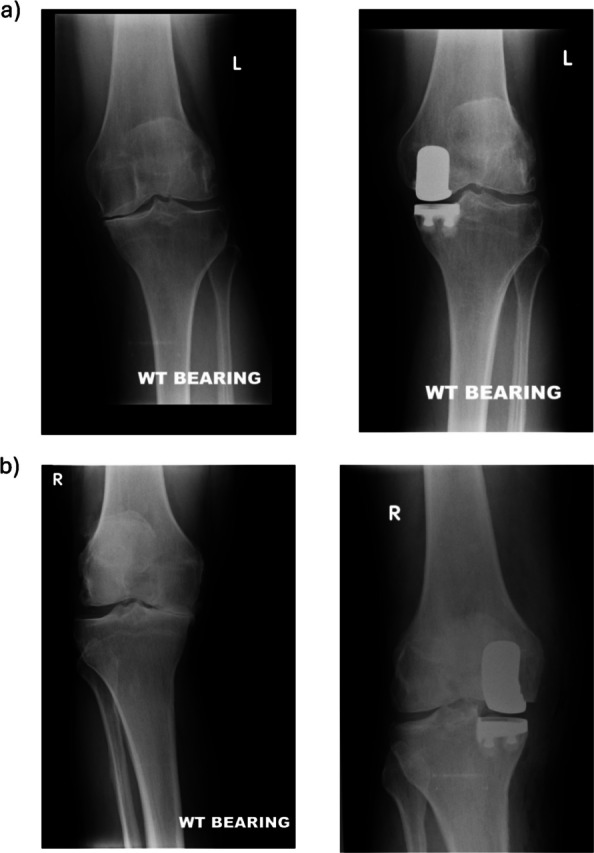
**a** Pre- and postoperative AP views of a mild OA knee. **b** Pre- and post-operative AP views of a severe OA knee

Patients lie supine with a tourniquet applied using single-side support, a footrest for deep flexion, and a second footrest for mid-flexion. The surgical approach followed a paramedial incision from the superomedial corner of the patella, extending to the tibial tuberosity, after which a minimal parapatellar arthrotomy, limited anteromedial release, and partial resection of the fat pad established access [16]. Tibiofemoral and patellofemoral arthritis were assessed intraoperatively using the Outerbridge classification [17]. Grades 0, I, and II indicated no or minimal chondral lesions, while Grades III and IV indicated significant chondral lesions. The status of the ACL was then assessed and recorded. Bony resections were performed using standard instrumentation provided by the manufacturer. The surgical technique allowed for the maintenance of some residual deformity to facilitate balancing and avoid overcorrection (Fig. 2). Varus deformity was assessed and deemed correctable if constitutional alignment could be achieved by a manual stress test with the knee in slight flexion. After the bone surfaces were prepared, the tibial and femoral components were cemented. Immediate full weight bearing was authorised, and rehabilitation began on the day of surgery.

### Patient-reported outcome measures (PROMs)

Before surgery, patients completed questionnaires for the Oxford Knee Score (OKS), the EuroQol-5D (EQ-5D), Knee Injury and Osteoarthritis Outcome Score Physical Function Short Form (KOOS-PS), University of California, Los Angeles (UCLA) activity score, and Forgotten Joint Score (FJS). Patients completed the same questionnaires, as well as their level of satisfaction (“Very dissatisfied,” “Dissatisfied,” “Satisfied,” and “Very satisfied”) at 3 weeks, 3, 6, 12, 24, and 60 months.

### Statistical analysis

Continuous variables were summarised as means, standard deviations (SD), ranges, medians, and interquartile ranges (IQR). Sub-group analyses included the comparison of PROMs in patients (i) with Grade III & IV versus Grade 0 to III medial/central patellofemoral arthritis, (ii) with ACL deficiency versus intact ACL, (iii) aged younger than 50, 50 to 59, 60 to 69, 70 to 79 and older than 80, and (iv) with a BMI < 30, 30 ≤ BMI < 35, 35 ≤ BMI < 40 and BMI ≥ 40 with Bonferroni correction. Shapiro–Wilk tests were used to assess the normality of distributions, and differences in continuous variables between sub-groups (i) and (ii) were evaluated using the student’s t-test for normal distributions or Wilcoxon rank sum tests (Mann–Whitney U test) for non-normal distributions. Differences between the age and BMI subgroups were assessed using the Kruskal–Wallis test. The effect size for differences in continuous variables between groups was expressed as mean differences (MD) with 95% confidence intervals (95% CI). Categorical variables were summarised as frequencies and proportions. Differences in categorical variables between sub-groups were assessed using Chi-squared tests. The effect size of differences in categorical variables between groups was expressed as odds ratios (OR with 95% CI). Finally, multivariable analyses were performed to evaluate the independent associations of age, BMI, patellofemoral arthritis, and ACL status with OKS, EQ-5D, KOOS-PS, UCLA activity score, and FJS, adjusting for sex. Considering the recommendation of Austin and Steyerberg of at least 10 subjects per variable, the sample size of 221 knees was deemed sufficiently powered [18]. All statistical analyses were performed using R Studio (2023.09.1 Build 494, Posit Software, PBC) and R (version 4.3.1, R Core Team (2023). R: A Language and Environment for Statistical Computing. R: Foundation for Statistical Computing, Vienna, Austria.). The authors used *ggpubr* [19], *reshape* [20], *meta* [21], and *openxlsx* [22] packages.

## Results

There were no statistically significant differences in OKS, EQ-5D, KOOS PS, FJS-12, or UCLA activities between the 2- and 5-year follow-up assessments. At 5-year follow-up, preoperative OKS improved from 24.9 ± 6.5 to 44.3 ± 5.0 (MD = 19.4; 95% CI = 18.3 to 20.5), preoperative EQ-5D improved from 0.5 ± 0.2 to 0.9 ± 0.1 (MD = 0.4; 95% CI = 0.38 to 0.43), preoperative KOOS PS improved from 55.2 ± 12.3 to 85.8 ± 14.3 (MD = 30.6; 95% CI = 28.1 to 33.1), preoperative FJS-12 from 15.3 ± 14.1 to 80.8 ± 22.6 (MD = 65.5; 95% CI = 62.0 to 69.0), and preoperative UCLA Activities from 4.8 ± 2.0 to 6.3 ± 1.8 (MD = 1.5; 95% CI = 1.1 to 1.9) (Table 2). Satisfaction rates of the entire cohort remained consistent at the 2- and 5-year follow-up points, with 96% being either satisfied or very satisfied, irrespective of their patellofemoral arthritis grade, ACL status, age, sex, or BMI.

**Table 2:** Overall patient-reported outcome measures (PROMs)

Variable	Mean ± SD	Range	Median (IQR)	*n* (%)	*p*-value (2 vs 5 yrs)
**Follow-up (years)**	6.1 ± 0.5	5.0–7.0	6.1 (5.6–6.5)		
**OKS**					0.114
Preoperative	24.9 ± 6.5	1–43	25 (21–29)		
2 years	44.7 ± 5.3	17–48	47 (44–48)		
5 years	44.3 ± 5.0	19–48	46 (43–48)		
**EQ-5D Index**					0.054
Preoperative	0.5 ± 0.2	0.0–0.8	0.6 (0.4–0.7)		
2 years	0.9 ± 0.1	0.1–1.0	1.0 (0.8–1.0)		
5 years	0.9 ± 0.1	0.2–1.0	1.0 (0.8–1.0)		
**KOOS PS**					0.237
Preoperative	55.2 ± 12.3	0.0–85.2	56.0 (48.8–63.0)		
2 years	84.1 ± 16.1	29.7–100.0	89.5 (75.1–100.0)		
5 years	85.8 ± 14.3	29.7–100.0	89.5 (78.0–100.0)		
**FJS-12**					0.865
Preoperative	15.3 ± 14.1	0.0–68.8	12.5 (5.0–22.5)		
2 years	80.5 ± 24.7	2.1–100.0	91.7 (70.9–97.9)		
5 years	80.8 ± 22.6	0.0–100.0	87.5 (70.8–100.0)		
**UCLA Activity Score**					0.306
Preoperative	4.8 ± 2.0	1–10	4 (3–6)		
2 years	6.5 ± 1.8	2–10	6 (5–8)		
5 years	6.3 ± 1.8	3–10	6 (5–8)		
**Satisfaction**					0.770
**2 years**					
Very satisfied				179 (81%)	
Satisfied				34 (15%)	
Dissatisfied				7 (3%)	
Very dissatisfied				0 (0%)	
**5 years**					
Very satisfied				182 (82%)	
Satisfied				31 (14%)	
Dissatisfied				5 (2%)	
Very dissatisfied				3 (1%)	

Values are presented as mean ± standard deviation (SD) or median (interquartile range [IQR]) for continuous variables, and as frequency (percentage) for categorical variables. The scoring ranges for the outcome measures are as follows, with higher scores indicating better outcomes: OKS (0–48), EQ-5D index (0–1.0), KOOS PS (0–100), FJS-12 (0–100), and UCLA Activity Score (0–10)

*Abbreviations:* OKS, Oxford Knee Score; KOOS PS, Knee Injury and Osteoarthritis Outcome Score Physical Function; FJS, Forgotten Joint Score; UCLA, University of California Los Angeles; sd, standard deviation; IQR, interquartile range

Pairwise comparisons between knees with Grade 0/I/II versus Grade III/IV central/medial patellofemoral arthritis revealed no statistically significant differences at five years for OKS (MD = 0.6; *p* = 0.419), EQ-5D (MD = 0.03; *p* = 0.100), KOOS PS (MD = 0.9; *p* = 0.848), FJS-12 (MD = 2.6; *p* = 0.848) or UCLA Activities (MD = 0.3; *p* = 0.921) (Table 3).

**Table 3:** Comparison of patient-reported outcome measures (PROMs) between knees with Grade 0/I/II and Grade III/IV medial/central patellofemoral arthritis

Outcome	Grade 0/I/II (*n* = 101)			Grade III/IV (*n* = 120)			Statistics		
	**Mean ± SD/*n* (%)**	**Median (IQR)**	**Range**	**Mean ± SD/*n* (%)**	**Median (IQR)**	**Range**	***p*-value**	**MD/OR**	**95% CI**
**OKS**									
Preoperative	24.8 ± 6.0	25 (20–29)	10–43	24.9 ± 6.8	26 (21–29)	1–43	0.923	− 0.1	− 1.8 to 1.6
2 years	45.1 ± 4.3	47 (45–47)	21–48	44.3 ± 6.1	47 (44–48)	17–48	0.465	0.8	− 0.6 to 2.2
5 years	44.6 ± 4.9	46 (43–48)	19–48	44.0 ± 5.2	46 (43–48)	23–48	0.419	0.6	− 0.8 to 1.9
**EQ-5D Index**									
Preoperative	0.5 ± 0.2	0.6 (0.4–0.7)	0.0–0.8	0.5 ± 0.2	0.5 (0.4–0.7)	0.0–0.8	0.427	0.03	− 0.02 to 0.09
2 years	0.9 ± 0.1	1.0 (0.8–1.0)	0.6–1.0	0.9 ± 0.2	1.0 (0.8–1.0)	0.1–1.0	0.735	0.03	− 0.01 to 0.06
5 years	0.9 ± 0.1	1.0 (0.8–1.0)	0.2–1.0	0.9 ± 0.1	1.0 (0.8–1.0)	0.5–1.0	0.100	0.03	− 0.01 to 0.06
**KOOS PS**									
Preoperative	56.7 ± 10.7	58.0 (51.5–64.7)	28.2–78.0	54.0 ± 13.4	53.9 (48.8–61.4)	0.0–85.2	0.104	2.7	− 0.5 to 5.9
2 years	84.3 ± 14.6	89.5 (77.7–94.4)	42.0–100.0	83.9 ± 17.3	89.5 (73.8–100.0)	29.7–100.0	0.650	0.4	− 3.8 to 4.7
5 years	86.2 ± 13.8	89.5 (78.0–100.0)	38.0–100.0	85.4 ± 14.8	89.5 (78.0–100.0)	29.7–100.0	0.848	0.9	− 2.9 to 4.6
**FJS-12**									
Preoperative	15.9 ± 13.5	12.5 (5.0–23.0)	0.0–62.5	14.9 ± 14.6	12.3 (4.9–20.9)	0.0–68.8	0.324	1.0	− 2.7 to 4.8
2 years	81.3 ± 23.8	91.7 (75.0–95.9)	2.1–100.0	79.8 ± 25.6	91.7 (66.7–100.0)	2.1–100.0	0.732	1.5	− 5.0 to 8.0
5 years	82.3 ± 20.9	90.0 (75.0–97.9)	8.3–100.0	79.7 ± 23.9	87.5 (70.8–100.0)	0.0–100.0	0.921	2.6	− 3.3 to 8.5
**UCLA Activities**									
Preoperative	4.8 ± 2.0	4 (3–6)	2–10	4.9 ± 2.1	4 (3–6)	1–10	0.919	0.0	− 0.6 to 0.5
2 years	6.5 ± 1.6	6 (6–8)	3–10	6.5 ± 1.9	6 (5–8)	2–10	0.845	0.0	− 0.4 to 0.5
5 years	6.5 ± 1.6	6 (6–8)	3–10	6.2 ± 1.9	6 (5–8)	3–10	0.226	0.3	− 0.2 to 0.7
**Satisfaction at 2 years**							0.828		
Very dissatisfied	0 (0%)			0 (0%)					
Dissatisfied	3 (3%)			4 (3%)				0.89	0.19 to 4.06
Satisfied	16 (16%)			18 (15%)				1.07	0.51 to 2.22
Very satisfied	82 (81%)			97 (81%)				1.02	0.52 to 2.01
**Satisfaction at 5 years**							0.734		
Very dissatisfied	1 (1%)			2 (2%)				0.59	0.05 to 6.60
Dissatisfied	3 (3%)			2 (2%)				1.81	0.30 to 11.03
Satisfied	12 (12%)			19 (16%)				0.72	0.33 to 1.56
Very satisfied	85 (84%)			97 (81%)				1.26	0.62 to 2.54

Values are presented as mean ± standard deviation, median (interquartile range [IQR]), and range for continuous variables, or as frequency (percentage) for categorical variables. The scoring ranges for the outcome measures are as follows, with higher scores indicating better outcomes: OKS (0–48), EQ-5D index (0–1.0), KOOS PS (0–100), FJS-12 (0–100), and UCLA Activity Score (0–10)

*Abbreviations:* OKS, Oxford Knee Score; KOOS PS, Knee Injury and Osteoarthritis Outcome Score Physical Function Short Form; FJS-12, Forgotten Joint Score-12; MD, mean difference; OR, odds ratio; CI, confidence interval

Pairwise comparisons between knees with intact versus deficient ACL revealed no statistically significant differences at five years for OKS (MD = 1.9; *p* = 0.099), EQ-5D (MD = 0.06; *p* = 0.109), KOOS PS (MD = 6.0; *p* = 0.113), FJS-12 (MD = 5.9; *p* = 0.180) or UCLA activities (MD = 0.0; *p* = 0.991) at the 5-year follow-up point (Table 4).

**Table 4:** Comparison of patient-reported outcome measures (PROMs) between knees with intact and deficient ACL

Outcome	Intact ACL (*n* = 201)			Deficient ACL (*n* = 20)			Statistics		
	**Mean ± SD/*n* (%)**	**Median (IQR)**	**Range**	**Mean ± SD/*n* (%)**	**Median (IQR)**	**Range**	***p*-value**	**MD/OR**	**95% CI**
**OKS**									
Preoperative	24.6 ± 6.6	25 (20–29)	1–43	27.5 ± 4.6	27 (25–29)	19–37	0.056	2.9	0.7 to 5.1
2 years	44.5 ± 5.5	47 (44–48)	17–48	46.7 ± 2.2	48 (46–48)	40–48	0.022	2.1	0.9 to 3.4
5 years	44.1 ± 5.2	46 (43–48)	19–48	46.1 ± 2.4	47 (46–48)	41–48	0.099	1.9	0.7 to 3.2
**EQ-5D Index**									
Preoperative	0.5 ± 0.2	0.6 (0.4–0.7)	0.0–0.8	0.6 ± 0.2	0.6 (0.5–0.7)	0.2–0.8	0.118	0.07	− 0.01 to 0.15
2 years	0.9 ± 0.1	1.0 (0.8–1.0)	0.1–1.0	1.0 ± 0.1	1.0 (1.0–1.0)	0.6–1.0	0.141	0.05	0.00 to 0.09
5 years	0.9 ± 0.1	1.0 (0.8–1.0)	0.2–1.0	1.0 ± 0.1	1.0 (1.0–1.0)	0.7–1.0	0.109	0.06	0.01 to 0.10
**KOOS PS**									
Preoperative	54.9 ± 12.5	56.0 (48.8–63.3)	0.0–85.2	58.7 ± 9.6	59.7 (54.3–63.0)	38.0–78.0	0.195	3.9	− 0.7 to 8.4
2 years	83.2 ± 16.4	89.5 (75.1–94.4)	29.7–100.0	92.3 ± 8.3	94.4 (85.0–100.0)	75.1–100.0	0.017	9.1	4.8 to 13.4
5 years	85.2 ± 14.7	89.5 (75.1–100.0)	29.7–100.0	91.2 ± 8.4	94.4 (84.3–100.0)	78.0–100.0	0.113	6.0	1.8 to 10.2
**FJS-12**									
Preoperative	14.7 ± 13.6	12.5 (5.0–21.0)	0.0–67.5	21.4 ± 17.5	13.8 (11.5–27.8)	0.0–68.8	0.064	6.7	− 1.2 to 14.6
2 years	79.4 ± 25.3	90.6 (70.4–97.9)	2.1–100.0	91.1 ± 13.9	97.8 (90.6–100.0)	54.2–100.0	0.013	11.7	4.7 to 18.8
5 years	80.3 ± 22.9	87.5 (70.8–100.0)	0.0–100.0	86.3 ± 18.5	91.7 (84.9–100.0)	43.8–100.0	0.180	5.9	− 2.7 to 14.6
**UCLA Activities**									
Preoperative	4.8 ± 2.1	4 (3–6)	1–10	5.4 ± 1.8	6 (4–6)	2–8	0.126	0.6	− 0.3 to 1.4
2 years	6.4 ± 1.8	6 (5–8)	2–10	7.0 ± 1.5	7 (6–8)	4–10	0.172	0.6	− 0.1 to 1.2
5 years	6.3 ± 1.8	6 (5–8)	3–10	6.4 ± 1.9	6 (5–8)	4–10	0.991	0.0	− 0.8 to 0.9
**Satisfaction at 2 years**							0.412		
Very dissatisfied	0 (0%)			0 (0%)					
Dissatisfied	7 (3%)			0 (0%)				1.58	0.09 to 28.69
Satisfied	33 (16%)			1 (5%)				3.73	0.48 to 28.85
Very satisfied	160 (80%)			19 (95%)				0.21	0.03 to 1.58
**Satisfaction at 5 years**							0.751		
Very dissatisfied	3 (1%)			0 (0%)				0.72	0.04 to 14.49
Dissatisfied	5 (2%)			0 (0%)				1.15	0.06 to 21.50
Satisfied	29 (14%)			2 (10%)				1.52	0.33 to 6.89
Very satisfied	164 (82%)			18 (90%)				0.49	0.11 to 2.22

*Notes:* Values are presented as mean ± standard deviation, median (interquartile range [IQR]), and range for continuous variables, or as frequency (percentage) for categorical variables. The scoring ranges for the outcome measures are as follows, with higher scores indicating better outcomes: OKS (0–48), EQ-5D index (0–1.0), KOOS PS (0–100), FJS-12 (0–100), and UCLA Activity Score (0–10)

*Abbreviations:* ACL, anterior cruciate ligament; OKS, Oxford Knee Score; KOOS PS, Knee Injury and Osteoarthritis Outcome Score Physical Function Short Form; FJS-12, Forgotten Joint Score-12; MD, mean difference; OR, odds ratio; CI, confidence interval

Comparisons between knees of different age groups revealed no differences at five years for OKS (*p* = 0.145), EQ-5D (*p* = 0.309), KOOS-PS (*p* = 0.117), or FJS-12 (*p* = 0.396). UCLA activities at the 5-year follow-up differed among patients aged 50 to 59 compared to those aged 80 and older (MD = 1.5; *p* = 0.027), patients aged 60 to 69 compared to those aged 70 to 79 (MD = 0.9; *p* = 0.014), and patients aged 60 to 69 compared to those aged 80 and older (MD = 1.7; *p* = 0.004). Rates of satisfaction at the 5-year follow-up was significantly different among patients younger than 50 compared to those aged 70 to 79 (90% vs 95% satisfied or very satisfied; *p* = 0.039), between patients aged 50 to 59 and those aged 60 to 69 (93% vs 100% satisfied or very satisfied; *p* < 0.001), and between patients aged 50 to 59 and those aged 70 to 79 (93% vs 95% satisfied or very satisfied; *p* = 0.039) (Table 5).

**Table 5:** Comparison of patient-reported outcome measures (PROMs) for patients of different ages

Outcome	< 50 years (*n* = 10)		50–59 years (*n* = 60)		60–69 years (*n* = 68)		70–79 years (*n* = 62)		≥ 80 years		Kruskal–Wallis test
	**Mean ± SD/*n* (%)**	**Range**	**Mean ± SD/*n* (%)**	**Range**	**Mean ± SD/*n* (%)**	**Range**	**Mean ± SD/*n* (%)**	**Range**	**Mean ± SD/*n* (%)**	**Range**	***p*-value**
**OKS**											
Preoperative	21.0 ± 5.4	9–30	24.2 ± 7.0	1–36	25.7 ± 6.3	11–43	25.7 ± 6.2	10–43	23.6 ± 5.7	15–36	0.156
2 years	44.1 ± 9.6	17–48	44.2 ± 5.0	29–48	45.5 ± 3.6	28–48	44.2 ± 6.5	20–48	45.0 ± 4.8	28–48	0.438
5 years	43.3 ± 7.5	23–48	43.6 ± 5.6	19–48	45.6 ± 2.7	34–48	43.9 ± 5.9	26–48	43.7 ± 4.4	29–48	0.145
**EQ-5D Index**											
Preoperative	0.5 ± 0.2	0.1–0.7	0.5 ± 0.2	0.0–0.8	0.5 ± 0.2	0.0–0.8	0.6 ± 0.2	0.1–0.8	0.5 ± 0.2	0.2–0.8	0.283
2 years	0.9 ± 0.3	0.1–1.0	0.9 ± 0.1	0.6–1.0	0.9 ± 0.1	0.6–1.0	0.9 ± 0.2	0.4–1.0	0.9 ± 0.1	0.6–1.0	0.476
5 years	0.9 ± 0.2	0.5–1.0	0.9 ± 0.2	0.2–1.0	0.9 ± 0.1	0.7–1.0	0.9 ± 0.1	0.5–1.0	0.9 ± 0.1	0.6–1.0	0.309
**KOOS PS**											
Preoperative	54.0 ± 14.3	22.3–75.1	56.5 ± 15.2	0.0–85.2	55.3 ± 10.4	28.2–75.1	54.3 ± 10.6	28.2–78.0	54.3–12.8	28.2–78.0	0.665
2 years	86.4 ± 20.7	31.8–100.0	82.1 ± 15.8	42.0–100.0	85.9 ± 14.2	48.0–100.0	82.7 ± 16.6	38.6–100.0	86.3–18.9	29.7–100.0	0.279
5 years	84.4 ± 13.2	58.0–100.0	85.2 ± 14.3	38.0–100.0	89.5 ± 11.3	59.7–100.0	82.7 ± 16.9	29.7–100.0	85.0 ± 13.6	59.7–100.0	0.177
**FJS-12**											
Preoperative	11.4 ± 10.9	0.0–35.5	15.5 ± 13.9	0.0–62.5	14.9 ± 13.7	0.0–56.3	15.4 ± 16.1	0.0–68.8	18.0 ± 11.8	2.5–43.8	0.501
2 years	82.0 ± 29.5	10.4–100.0	79.6 ± 23.1	14.6–100.0	79.1 ± 26.0	4.8–100.0	80.0 ± 25.7	2.1–100.0	87.9 ± 20.0	29.2–100.0	0.237
5 years	79.3–25.5	14.6–100.0	76.4 ± 25.0	8.3–100.0	86.0 ± 16.5	43.8–100.0	79.9 ± 24.1	0.0–100.0	80.3 ± 24.9	14.6–100.0	0.396
**UCLA Activities**											
Preoperative	3.9 ± 2.0	2–8	4.7 ± 2.1	1–10	5.4 ± 2.1	2–10	4.9 ± 1.8	2–10	4.0 ± 1.9	2–9	0.101
2 years	6.1 ± 2.1	3–10	6.7 ± 1.7	3–10	7.0 ± 1.6	3–10	6.0 ± 1.9	2–10	5.9 ± 1.6	3–10	0.002
5 years	6.0 ± 2.3	3–10	6.6 ± 1.7	3–10	6.8 ± 1.5	3–10	5.9 ± 1.7	3–10	5.1 ± 1.9	3–8	< 0.001
**Satisfaction at 2 years**											
Very dissatisfied	0 (0%)		0 (0%)		0 (0%)		0 (0%)		0 (0%)		0.358
Dissatisfied	1 (10%)		2 (3%)		0 (0%)		4 (6%)		0 (0%)		
Satisfied	2 (20%)		9 (15%)		12 (18%)		10 (16%)		1 (5%)		
Very satisfied	7 (70%)		49 (82%)		56 (82%)		48 (77%)		19 (90%)		
**Satisfaction at 5 years**											
Very dissatisfied	0 (0%)		0 (0%)		0 (0%)		3 (5%)		0 (0%)		0.006
Dissatisfied	1 (10%)		4 (7%)		0 (0%)		0 (0%)		0 (0%)		
Satisfied	0 (0%)		15 (25%)		5 (7%)		8 (13%)		3 (14%)		
Very satisfied	9 (90%)		41 (68%)		63 (93%)		51 (82%)		18 (86%)		
**Pairwise comparisons**											
**Outcome**											
**UCAL activities**		50–59		60–69		70–79		> 80			
2 years	< 50	1.000		0.824		1.000		1.000			
	50–59			1.000		0.205		0.197			
	60–69					0.011		0.005			
	70–79							1.000			
5 years	< 50	1.000		0.704		1.000		1.000			
	50–59			1.000		0.212		0.027			
	60–69					0.014		0.004			
	70–79							0.430			
**Satisfaction**		50–59		60–69		70–79		> 80			
5 years	< 50	50–59		60–69		70–79		> 80			
	50–59			1.000		0.212		0.027			
	60–69					0.014		0.004			
	70–79							0.430			

*Notes:* Values are presented as mean ± standard deviation, and range for continuous variables, or as frequency (percentage) for categorical variables. The scoring ranges for the outcome measures are as follows, with higher scores indicating better outcomes: OKS (0–48), EQ-5D index (0–1.0), KOOS PS (0–100), FJS-12 (0–100), and UCLA Activity Score (0–10)

*Abbreviations:* ACL, anterior cruciate ligament; OKS, Oxford Knee Score; KOOS PS, Knee Injury and Osteoarthritis Outcome Score Physical Function Short Form; FJS-12, Forgotten Joint Score-12

Comparisons between knees of different BMI groups revealed no differences at five years for OKS (*p* = 0.170), EQ-5D (*p* = 0.622), KOOS-PS (*p* = 0.121), or FJS-12 (*p* = 0.793). UCLA activities at the 5-year follow-up differed between patients with a BMI < 30 and those with a BMI ≥ 40 (MD = 2.3; *p* = 0.045) (Table 6).

**Table 6:** Comparison of patient-reported outcome measures (PROMs) for patients with different body mass index (BMI)

Outcome	BMI < 30 (*n* = 134)		30 ≤ BMI < 35 (*n* = 65)		35 ≤ BMI < 40 (*n* = 18)		BMI ≥ 40 (*n* = 4)		Kruskall Wallace test
	**Mean ± SD/*n* (%)**	**Range**	**Mean ± SD/*n* (%)**	**Range**	**Mean ± SD/*n* (%)**	**Range**	**Mean ± SD/*n* (%)**	**Range**	***p*-value**
**OKS**									
Preoperative	25.5 ± 6.6	6–43	24.2 ± 5.6	12–38	23.7 ± 7.9	1–36	19.5 ± 3.4	16–24	0.088
2 years	44.8 ± 5.4	17–48	44.8 ± 5.1	20–48	44.4 ± 5.3	30–48	41.3 ± 9.0	29–48	0.960
5 years	44.7 ± 4.8	23–48	43.9 ± 5.3	19–48	43.5 ± 4.3	31–48	39.5 ± 9.3	31–48	0.170
**EQ-5D Index**									
Preoperative	0.6 ± 0.2	0.0–0.8	0.5 ± 0.2	0.0–0.8	0.5 ± 0.2	0.0–0.8	0.3 ± 0.2	0.3–0.8	0.035
2 years	0.9 ± 0.1	0.1–1.0	0.9 ± 0.1	0.4–1.0	0.9 ± 0.1	0.7–1.0	0.8 ± 0.2	0.6–1.0	0.717
5 years	0.9 ± 0.1	0.5–1.0	0.9 ± 0.2	0.2–1.0	0.9 ± 0.1	0.6–1.0	0.8 ± 0.2	0.6–1.0	0.622
**KOOS PS**									
Preoperative	55.4 ± 12.3	0.0–85.2	55.3 ± 10.3	28.2–78.0	53.2 ± 18.1	0.0–78.0	55.7 ± 13.9	42.1–75.1	0.958
2 years	84.4 ± 15.7	29.7–100.0	84.8 ± 16.1	38.6–100.0	80.7 ± 16.0	42.0–100.0	76.3 ± 28.7	42.0–100.0	0.649
5 years	87.3 ± 13.3	45.6–100.0	85.2 ± 13.8	38.0–100.0	80.6 ± 17.2	29.7–100.0	68.2 ± 26.2	41.0–100.0	0.121
**FJS-12**									
Preoperative	16.2 ± 14.9	0.0–68.8	14.8 ± 13.6	0.0–67.5	12.3 ± 11.2	0.0–45.0	7.5 ± 3.8	4.2–12.5	0.502
2 years	80.1 ± 24.4	2.1–100.0	82.4 ± 22.8	8.3–100.0	79.5 ± 23.2	16.7–100.0	65.6 ± 41.0	18.8–100.0	0.828
5 years	80.5–23.0	0.0–100.0	72.9 ± 20.1	8.3–100.0	80.0 ± 23.4	16.7–100.0	62.0 ± 39.4	23.1–100.0	0.793
**UCLA Activities**									
Preoperative	5.2 ± 2.1	2–10	4.4 ± 1.8	2–9	4.0 ± 1.8	1–8	3.0 ± 0.8	2–4	0.003
2 years	6.7 ± 1.9	3–10	6.2 ± 1.6	2–9	5.9 ± 1.6	3–9	5.3 ± 1.0	4–6	0.069
5 years	6.6 ± 1.8	3–10	5.9 ± 1.6	3–9	6.1 ± 1.8	3–9	4.3 ± 0.5	4–5	0.003
**Satisfaction at 2 years**									
Dissatisfied	5 (4%)		1 (2%)		1 (6%)		0 (0%)		0.304
Satisfied	22 (16%)		8 (12%)		2 (11%)		2 (50%)		
Very satisfied	106 (79%)		56 (86%)		15 (83%)		2 (50%)		
**Satisfaction at 5 years**									
Very dissatisfied	2 (1%)		0 (0%)		1 (6%)		0 (0%)		
Dissatisfied	3 (2%)		1 (2%)		0 (0%)		1 (25%)		
Satisfied	19 (14%)		8 (12%)		3 (17%)		1 (25%)		
Very satisfied	110 (82%)		56 (86%)		14 (78%)		2 (50%)		
**Pairwise comparisons**									
**Outcome**									
**UCAL activities**		30 ≤ BMI < 35		35 ≤ BMI < 40		BMI > 40			
5years	BMI < 30	0.045		0.440		0.045			
	30 ≤ BMI < 35			0.783		0.157			
	35 ≤ BMI < 40					0.198			

*Notes:* Values are presented as mean ± standard deviation, and range for continuous variables, or as frequency (percentage) for categorical variables. The scoring ranges for the outcome measures are as follows, with higher scores indicating better outcomes: OKS (0–48), EQ-5D index (0–1.0), KOOS PS (0–100), FJS-12 (0–100), and UCLA Activity Score (0–10)

*Abbreviations:* ACL, anterior cruciate ligament; OKS, Oxford Knee Score; KOOS PS, Knee Injury and Osteoarthritis Outcome Score Physical Function Short Form; FJS-12, Forgotten Joint Score-12; BMI, body mass index

Multivariable regression showed that increasing age was associated with worse UCLA activity scores (β =  − 0.028; *p* = 0.041), while men had higher UCLA activity scores (+ 0.93 points; β = 0.931; *p* < 0.001). Higher BMI was associated with worse OKS (β =  − 0.228; *p* = 0.017), KOOS-PS (β =  − 0.914; *p* < 0.001), and UCLA activity scores (β =  − 0.073; *p* = 0.023). Grade III/IV patellofemoral arthritis was associated with worse EQ-5D scores (β =  − 0.047; *p* = 0.045), while ACL deficiency was associated with higher KOOS-PS scores (β = 7.957; *p* = 0.023) (Table 7).

**Table 7:** Multivariable analysis to correct for sex and estimate the interaction effects of age, BMI, patellofemoral arthritis, and ACL deficiency

Outcome	Confounder	β/OR	95% CI	*p*-value
**OKS**				
	Age, years	− 0.029	− 0.109 to 0.051	0.477
	Men (*n* = 128)	1.066	− 0.500 to 2.633	0.181
	BMI, kg/m^2^	− 0.228	− 0.415 to − 0.041	0.017
	Grade III/IV central/medial patellofemoral arthritis (*n* = 120)	− 0.994	− 2.562 to 0.573	0.212
	Knees with a deficient ACL (*n* = 20)	2.423	− 0.126 to 4.971	0.062
**EQ-5D Index**				
	Age, years	5.67 × 10⁻^5^	− 2.29 × 10⁻^3^ to 2.40 × 10⁻^3^	0.962
	Men (*n* = 128)	3.02 × 10⁻^3^	− 4.29 × 10⁻^2^ to 4.89 × 10⁻^2^	0.897
	BMI, kg/m^2^	− 4.76 × 10⁻^3^	− 1.02 × 10⁻^2^ to 6.96 × 10⁻^4^	0.087
	Grade III/IV central/medial patellofemoral arthritis (*n* = 120)	− 4.69 × 10⁻^2^	− 9.29 × 10⁻^2^ to − 1.02 × 10⁻^3^	0.045
	Knees with a deficient ACL (*n* = 20)	6.39 × 10⁻^2^	− 1.14 × 10⁻^2^ to 1.37 × 10⁻^2^	0.097
**KOOS PS**				
	Age, years	− 0.043	− 0.259 to 0.173	0.693
	Men (*n* = 128)	3.928	− 0.283 to 8.139	0.067
	BMI, kg/m^2^	− 0.914	− 1.416 to − 0.411	< 0.011
	Grade III/IV central/medial patellofemoral arthritis (*n* = 120)	− 3.801	− 8.015 to 0.413	0.077
	Knees with a deficient ACL (*n* = 20)	7.957	1.106 to 14.807	0.023
**FJS-12**				
	Age, years	− 0.018	− 0.398 to 0.362	0.952
	Men (*n* = 128)	4.351	− 3.057 to 11.759	0.248
	BMI, kg/m^2^	− 0.445	− 1.329 to 0.440	0.322
	Grade III/IV central/medial patellofemoral arthritis (*n* = 120)	− 4.535	− 11.948 to 2.878	0.229
	Knees with a deficient ACL (*n* = 20)	5.459	− 6.593 to 17.510	0.372
**UCLA Activities**				
	Age, years	− 0.028	− 0.060 to − 0.001	0.041
	Men (*n* = 128)	0.931	0.400 to 1.458	< 0.001
	BMI, kg/m^2^	− 0.073	− 0.140 to − 0.010	0.023
	Grade III/IV central/medial patellofemoral arthritis (*n* = 120)	− 0.069	− 0.600 to 0.458	0.796
	Knees with a deficient ACL (*n* = 20)	0.483	− 0.370 to 1.340	0.268
**Satisfaction**				
	Age, years	0.982	0.856 to 1.110	0.775
	Men (*n* = 128)	0.410	0.018 to 4.719	0.483
	BMI, kg/m^2^	1.122	0.875 to 1.402	0.315
	Grade III/IV central/medial patellofemoral arthritis (*n* = 120)	2.410	0.209 to 57.935	0.496
	Knees with a deficient ACL (*n* = 20)	Not applicable*		

^*^For knees with a deficient ACL, no events of dissatisfaction occurred, resulting in complete separation in the logistic regression model. Consequently, odds ratios and confidence intervals for this variable could not be reliably estimated using standard maximum likelihood methods

*Notes:* The scoring ranges for the outcome measures are as follows, with higher scores indicating better outcomes: OKS (0–48), EQ-5D index (0–1.0), KOOS PS (0–100), FJS-12 (0–100), and UCLA Activity Score (0–10)

*Abbreviations:* ACL, anterior cruciate ligament; OKS, Oxford Knee Score; KOOS PS, Knee Injury and Osteoarthritis Outcome Score Physical Function Short Form; FJS-12, Forgotten Joint Score-12; MD, mean difference; OR, odds ratio; CI, confidence interval; BMI, body mass index

## Discussion

The most important finding of this study revealed that fixed-bearing medial UKA granted excellent outcomes at five years for patients with end-stage medial tibiofemoral arthritis, irrespective of age or BMI, and even in knees with significant medial and central patellofemoral arthritis and functionally stable ACL deficiency. This finding supports the hypothesis that PROMs are unaffected by substantial medial/central patellofemoral arthritis, functionally stable ACL deficiency, age, or BMI.

Of the initial cohort of 229 patients (240 knees), only four knees (1.7%) required revision. This compares favourably to the 2025 National Joint Registry cumulative revision rate of fixed-bearing UKA: 3.18% for all fixed medial UKA and 1.88% for the Persona Partial Knee at 5 years. Revision rates after UKA are closely related to surgeon volume, with higher UKA usage associated with improved survivorship and PROMs [23]. Overly restrictive selection criteria reduce the number of eligible patients and may limit surgeon experience, whereas using UKA for bone-on-bone unicompartmental disease with a functionally preserved remainder of the knee allows adequate case volume without compromising outcomes [24]. In this context, accurate assessment of full-thickness cartilage loss is crucial for patient selection. A recent study demonstrated that while 44% of medial UKA candidates were graded as having less severe osteoarthritis (Kellgren–Lawrence 1–3) on radiographs, magnetic resonance imaging (MRI) evaluation revealed grade 4 ICRS cartilage loss in all cases, confirming eligibility for UKA and underscoring the importance of advanced imaging when radiographs are inconclusive [25].

The present study’s findings corroborate other studies that propose patellofemoral arthritis should not be considered a contraindication for medial UKA. A recent study on 81 medial UKAs found that patellofemoral arthritis did not affect clinical and functional outcomes, complication rates, or implant survival at the six-year follow-up [1]. Likewise, a recent prospective study on 678 medial UKAs revealed no association between function and patellofemoral arthritis at one-year follow-up [12]. Pre-existent patellofemoral arthritis, except for severe lateral osteoarthritis, can be ignored as a contraindication, even if assessed using MRI [25], as it does not impact postoperative survival or clinical outcomes in patients undergoing medial UKA [5].

Knees with arthritis and ACL deficiency appear in two patient groups [6]: (1) Secondary arthritis arises from an ACL rupture in typically younger, more active individuals; (2) An ACL tear occurs due to primary degenerative arthritis in generally older, less active patients. A recent study proposed that patients with ACL-deficient arthritis may still qualify for a UKA if the knee is functionally stable [6]. A previous study comparing survivorship, causes of reoperation, and functional outcomes of medial mobile-bearing UKA in patients with prior anterior cruciate ligament reconstruction (ACLr) and matched controls found a significantly higher reoperation rate and inferior implant survivorship in the ACLr group [26]. These patients also had an increased risk of subsequent surgery for bearing dislocation, a risk that is mitigated with fixed-bearing implants.

Indications for medial UKA have evolved over four decades, since only 2% to 12% of patients meet the classical Kozinn and Scott criteria published in 1989 [12]. Kozinn and Scott recommended restricting UKA usage to non-active patients aged 60 and above and weighing under 82 kg [5]. The present study included patients aged 41 to 89 years, with a BMI between 20 and 47 kg/m^2^. A recent meta-analysis reported no associations between younger age and higher revision rates or lower functional scores [27], suggesting that age alone should not be a contraindication for fixed-bearing medial UKA. Obesity is associated with higher rates of revision and complications after UKA, but the resulting reductions in functional outcomes are small and unlikely to be clinically meaningful; therefore, obesity alone should not be considered a contraindication [28][29, 30]. The present study included many patients with high UCLA activity scores (50% with UCLA activity > 6). Older and higher-BMI patients were less active, as confirmed by multivariable analysis, but only BMI was negatively associated with other PROMs. These findings could suggest that higher preoperative activity levels do not adversely impact mid-term outcomes and therefore should not be considered a contraindication to fixed-bearing medial UKA.

The present study’s findings should be interpreted considering the following factors and limitations. This is a single-series study from a surgeon with a high annual caseload of medial fixed-bearing UKA. Patellofemoral arthritis was graded intraoperatively using the Outerbridge classification, without pre- or post-operative imaging assessment, thereby limiting the reproducibility of the grading method by other research groups or monitoring progression. Univariate analysis (pairwise comparisons) did not demonstrate a statistically significant difference in EQ-5D scores between Grade III/IV and Grade 0–II patellofemoral arthritis groups. In contrast, the multivariable regression analysis identified an independent association after adjustment for age, sex, BMI, and ACL deficiency as covariates. This discrepancy likely reflects the influence of confounding factors and reduced residual variability in the adjusted model, which enabled more precise estimation of the independent effect of patellofemoral arthritis. Importantly, although the association was statistically significant, the magnitude of the regression coefficient was small and did not meet the threshold for clinical relevance. The study included a small subgroup of patients with ACL deficiency (*n* = 20), which may limit the statistical power to detect meaningful differences. Positive results amongst ACL-deficient patients should be considered cautiously, as this may reflect different patient expectations. The distribution of knees between age groups and BMI categories was unequal; therefore, outliers could influence the findings. Finally, while previous studies have examined the influence of BMI [29, 31, 32], age [7, 32], patellofemoral arthritis [14], and ACL status [14] on UKA outcomes, this study uniquely evaluates their independent impact on five-year patient-reported outcomes within a single multivariable analysis, providing clinically relevant insight into longer-term outcomes.

## Conclusion

Five-year outcomes following fixed-bearing medial UKA demonstrated high patient satisfaction, unchanged from two years. Although older age was associated with lower activity and higher BMI (> 40) with worse function, the effect sizes were small and not clinically meaningful. Patellofemoral arthritis and ACL deficiency had no negative functional impact. Therefore, age, BMI, patellofemoral arthritis, and ACL status should not be considered contraindications; instead, broad selection criteria for fixed-bearing UKA are supported.

## Data Availability

Upon reasonable request, the authors will consider making data available.
